# Immune adaptation to chronic intense exercise training: new microarray evidence

**DOI:** 10.1186/s12864-016-3388-5

**Published:** 2017-01-05

**Authors:** Dongmei Liu, Ru Wang, Ana R. Grant, Jinming Zhang, Paul M. Gordon, Yuqin Wei, Peijie Chen

**Affiliations:** 1 School of Kinesiology, Shanghai University of Sport, Qinyuanhuan Road, #650, Yangpu District, Shanghai, China; 2 Department of Computational Medicine & Bioinformatics / Bioinformatics Core, University of Michigan Medical School, Ann Arbor, MI USA; 3 College of sports medicine and rehabilitation, Taishan Medical University, Shandong Province, China; 4 Department of Health, Human Performance and Recreation, Baylor University, Waco, TX USA

**Keywords:** Transcriptome, Athletes, Immunity, Leukocytes, High-intensity endurance training

## Abstract

**Background:**

Endurance exercise training, especially the high-intensity training, exhibits a strong influence on the immune system. However, the mechanisms underpinning the immune-regulatory effect of exercise remain unclear. Consequently, we chose to investigate the alterations in the transcriptional profile of blood leukocytes in young endurance athletes as compared with healthy sedentary controls, using Affymetrix human gene 1.1 ST array.

**Results:**

Group differences in the transcriptome were analyzed using Intensity-based Hierarchical Bayes method followed by a Logistic Regression-based gene set enrichment method.

We identified 72 significant transcripts differentially expressed in the leukocyte transcriptome of young endurance athletes as compared with non-athlete controls with a false discovery rate (FDR) < 0.05, comprising mainly the genes encoding ribosomal proteins and the genes involved in mitochondrial oxidative phosphorylation. Gene set enrichment analysis identified three major gene set clusters: two were up-regulated in athletes including gene translation and ribosomal protein production, and mitochondria oxidative phosphorylation and biogenesis; one gene set cluster identified as transcriptionally downregulated in athletes was related to inflammation and immune activity.

**Conclusion:**

Our data indicates that in young healthy individuals, intense endurance exercise training (exemplifed by athletic training) can chronically induce transcriptional changes in the peripheral blood leukocytes, upregulating genes related to protein production and mitochondrial energetics, and downregulating genes involved in inflammatory response. The findings of the study also provide support for the notion that peripheral blood can be used as a surrogate tissue to study the systemic effect of exercise training.

**Electronic supplementary material:**

The online version of this article (doi:10.1186/s12864-016-3388-5) contains supplementary material, which is available to authorized users.

## Background

Endurance exercise training exhibits a powerful influence on the immune system. As a physical stressor, exercise can induce different immune responses, depending on the intensity and the duration of the exercise. They can be immunoprotective (e.g. enhancing wound healing and vaccination responses), immunopathological (e.g. increasing allergic or autoimmune responses) or immunoregulatory/inhibitory (e.g. anti-inflammatory effect) [1, 2]. It is generally believed that moderate intensity exercise can enhance immune function and reduce the risk of upper respiratory tract infection. Conversely, prolonged bouts of strenuous exercise can result in a transient depression of immune function [2, 3], which suggests that to protect immune function, individuals should avoid strenuous exercise. Nevertheless, emerging evidence suggests that high-intensity training can induce a more advanced anti-inflammatory response [4] which is favored in the prevention and treatment of diseases associated with chronic inflammation such as cardiovascular disease. Recently, it has been reported that former elite athletes with a history of vigorous physical activity had better metabolic health in later life than their controls, and this was independent of the effect of their current leisure-time physical activity levels [5]. For this reason, it has been suggested that increased susceptibility to minor infection is the small price to be paid for the long-term health benefits of regular exercise at high intensity [6].

To promote more effective use of high-intensity endurance exercise training in health promotion and disease prevention, a complete understanding of the nature of its immune regulatory effect is required. However, this is currently lacking. Immune response and regulation is complex involving a complicated interaction of a variety of immune cells, various cytokines, and chemokines. It has been widely accepted that assessing changes in transcript abundance in blood on a genome-wide scale, via transcript profiling using microarrays, affords a comprehensive view of the status of the immune system in health and disease, because leukocytes present in the blood convey valuable information about the status of the immune system [7]. Microarray has been used to study peripheral blood leukocytes in response to exercise [8–10]. However, the majority of studies have been undertaken to investigate the immune response to acute exercise in either the trained [8] or untrained state [9, 10]. Less is known concerning the alterations in the transcriptional profile of leukocytes induced by chronic high-intensity endurance exercise training, although it has been demonstrated that training influences the effect of acute exercise on immune cells [11]. This may happen because the change in immunity that occurs after each prolonged exercise bout is more clinically significant than training-induced alterations at rest in athletes. Consequently, to produce an unbiased global view of the primary and secondary molecular and cellular processes associated with the immune response to chronic high-intensity exercise training, we used young endurance athletes as high-intensity endurance exercise training model, analyzing their blood transcriptomic changes as compared with sedentary controls, using genome-wide microarray. Specifically, our aims were to gain a more complete understanding of the mechanism underlying the altered immunity, and to reveal molecular changes signifying latent immunological consequences as a result of regular exercise at high intensity.

Microarray data from the present study provided transcriptional evidence for the anti-inflammatory effect of high-intensity endurance exercise training, and produced novel data suggestive of immune enhancing effect of high-intensity endurance exercise training mediated by transcriptional upregulation of leukocyte mitochondrial energetics and ribosomal protein production.

## Results

### Physiological characteristics of subjects

Twelve endurance athletes and 12 healthy sedentary volunteers were included in the study. The athletes and non-athlete controls were group-wise matched for age, gender and BMI. The immune function markers in blood showed no significant difference between athletes and controls except for interleukin 1 receptor antagonist (IL-1ra), which was significantly lower in athletes (Table 1).

**Table 1:** Characteristics of the subjects

	Athletes (*n* = 12)	Non-athlete controls (*n* = 12)
Age (years)	18.4 ± 1.0	19.1 ± 1.1
BMI (kg/m^2^)	20.3 ± 1.8	20.6 ± 2.0
Gender	50% Female	50% Female
IgA (g/l)	2.0 ± 0.86	2.23 ± 0.96
IgM (g/l)	1.37 ± 0.52	1.44 ± 0.44
IgG (g/l)	11.92 ± 1.47	12.31 ± 2.31
IL-1ra (pg/ml)	242.37 ± 79.2^a^	480.56 ± 243.9

Values are expressed as mean ± sd; ^a^ Significantly different from controls

### Genes with differential expression in leukocytes in athletes

**Fig. 1:**
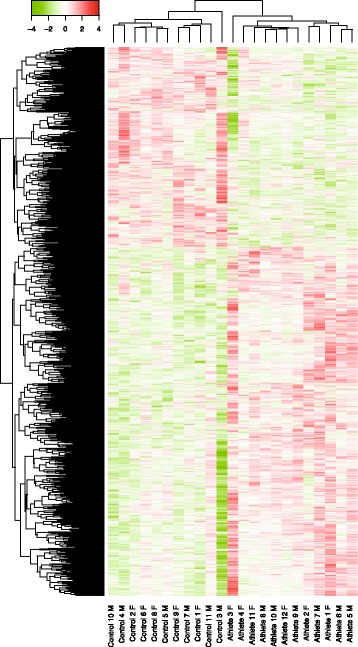
Heatmap of differential gene expression in young endurance athletes as compared with non-athlete controls. 2,658 genes with IBMT *p*-values ≤ 0.05 are included. Green represents downregulation of transcripts and red represents upregulation. Each column represents each subject and their identity (athlete vs. control, Female vs. male) is marked on the bottom of the map

Results of an intensity-based Bayesian moderated t-test (IBMT) revealed extensive transcriptional differences between athletes and controls across the leukocyte genome (Fig. 1). At the significance level of *p* < 0.05, 1723 genes exhibited higher transcript levels and 984 genes lower transcript levels in athletes compared with controls. The magnitude of change across the transcriptome was generally moderate. Using a stringent significance level of FDR < 0.05, 72 genes showed a differential expression with a mean fold change of 1.34 ± 0.13(mean ± std). The majority of them were up-regulated in athletes (70 genes) and only two were down-regulated. The information about these 72 genes is included in Table 2. The upregulated genes included mainly the genes encoding ribosomal proteins, and the genes involved in mitochondrial oxidative phosphorylation (OXPHOS).

**Table 2:** Significantly differentially expressed genes in young endurance athletes as compared with non-athlete controls

Gene Symbol	FC	ibmt *p*-value	FDR	Gene Description
SNORD14E	2.00	2.02E-05	2.02E-02	small nucleolar RNA, C/D box 14E
CKS2	1.82	4.56E-06	1.65E-02	CDC28 protein kinase regulatory subunit 2
SNORD4B	1.74	3.89E-06	1.65E-02	small nucleolar RNA, C/D box 4B
HLA-DPB1	1.58	4.40E-05	2.29E-02	major histocompatibility complex, class II, DP beta 1
ATP5G1	1.53	3.28E-05	2.02E-02	ATP synthase, H+ transporting, mitochondrial Fo complex, subunit C1
DDT	1.51	6.90E-05	3.09E-02	D-dopachrome tautomerase
TMEM141	1.51	3.71E-05	2.13E-02	transmembrane protein 141
BST2	1.50	1.39E-04	3.85E-02	bone marrow stromal cell antigen 2
LSM3	1.49	2.79E-05	2.02E-02	LSM3 homolog, U6 small nuclear RNA associated (S. cerevisiae)
COX7B	1.49	1.35E-04	3.78E-02	cytochrome c oxidase subunit VIIb
RPL10A	1.49	1.12E-04	3.78E-02	ribosomal protein L10a
RPL21	1.46	9.21E-05	3.74E-02	ribosomal protein L21
HNRNPC	1.45	2.48E-05	2.02E-02	heterogeneous nuclear ribonucleoprotein C (C1/C2)
MLLT4	1.45	1.77E-04	4.46E-02	myeloid/lymphoid or mixed-lineage leukemia
RPS27	1.44	1.12E-04	3.78E-02	ribosomal protein S27
RPS19	1.43	2.29E-04	4.70E-02	ribosomal protein S19
MRPL51	1.42	4.08E-05	2.20E-02	mitochondrial ribosomal protein L51
FASTKD3	1.42	2.00E-04	4.52E-02	FAST kinase domains 3
RPLP0	1.41	2.17E-04	4.69E-02	ribosomal protein, large, P0
MIR15B	1.41	1.89E-04	4.46E-02	microRNA 15b
UQCR10	1.41	1.10E-06	1.15E-02	ubiquinol-cytochrome c reductase, complex III subunit X
RAB33A	1.40	5.50E-06	1.65E-02	RAB33A, member RAS oncogene family
RPL23	1.40	6.30E-05	2.96E-02	ribosomal protein L23
EDF1	1.40	3.02E-05	2.02E-02	endothelial differentiation-related factor 1
RPL36A	1.40	2.25E-04	4.70E-02	ribosomal protein L36a
RPL31	1.38	1.31E-04	3.78E-02	ribosomal protein L31
RPS18P9	1.38	1.29E-04	3.78E-02	ribosomal protein S18 pseudogene 9
CD79B	1.38	2.72E-05	2.02E-02	CD79b molecule, immunoglobulin-associated beta
RPL6	1.37	6.25E-06	1.65E-02	ribosomal protein L6
SEC61G	1.37	1.08E-04	3.78E-02	Sec61 gamma subunit
SF3B5	1.37	8.80E-06	1.65E-02	splicing factor 3b, subunit 5, 10 kDa
TPI1	1.37	8.13E-06	1.65E-02	triosephosphate isomerase 1
MRPL28	1.37	2.08E-06	1.15E-02	mitochondrial ribosomal protein L28
ACOT13	1.36	2.26E-05	2.02E-02	acyl-CoA thioesterase 13
SNHG12	1.35	2.28E-04	4.70E-02	small nucleolar RNA host gene 12 (non-protein coding)
C15orf57	1.35	3.05E-05	2.02E-02	chromosome 15 open reading frame 57
BEX4	1.35	1.24E-04	3.78E-02	brain expressed, X-linked 4
TMEM223	1.34	9.57E-05	3.74E-02	transmembrane protein 223
PSMG3	1.34	1.12E-05	1.65E-02	proteasome (prosome, macropain) assembly chaperone 3
MZT2A	1.34	2.25E-04	4.70E-02	mitotic spindle organizing protein 2A
LIPT1	1.34	9.42E-05	3.74E-02	lipoyltransferase 1
ATP5J	1.33	1.93E-06	1.15E-02	ATP synthase, H+ transporting, mitochondrial Fo complex
HSF5	1.33	1.32E-04	3.78E-02	heat shock transcription factor family member 5
CCDC72	1.33	3.52E-05	2.11E-02	coiled-coil domain containing 72
RPP21	1.32	8.65E-05	3.69E-02	ribonuclease P/MRP 21 kDa subunit
THYN1	1.32	1.09E-05	1.65E-02	thymocyte nuclear protein 1
SNRPG	1.32	1.17E-04	3.78E-02	small nuclear ribonucleoprotein polypeptide G
C1orf220	1.32	1.20E-04	3.78E-02	chromosome 1 open reading frame 220
SSBP2	1.31	3.20E-05	2.02E-02	single-stranded DNA binding protein 2
DPY30	1.31	1.10E-05	1.65E-02	dpy-30 homolog (C. elegans)
COX4I1	1.31	1.89E-06	1.15E-02	cytochrome c oxidase subunit IV isoform 1
C14orf2	1.31	8.66E-05	3.69E-02	chromosome 14 open reading frame 2
C13orf27	1.30	6.09E-05	2.96E-02	chromosome 13 open reading frame 27
C6orf226	1.30	1.89E-04	4.46E-02	chromosome 6 open reading frame 226
NDUFA12	1.30	6.42E-05	2.96E-02	NADH dehydrogenase (ubiquinone) 1 alpha subcomplex, 12
MRPS11	1.30	2.28E-05	2.02E-02	mitochondrial ribosomal protein S11
CCDC56	1.30	2.05E-05	2.02E-02	coiled-coil domain containing 56
NDUFA2	1.30	1.77E-04	4.46E-02	NADH dehydrogenase (ubiquinone) 1 alpha subcomplex, 2, 8 kDa
PFDN1	1.30	1.82E-04	4.46E-02	prefoldin subunit 1
PDE6D	1.30	2.69E-05	2.02E-02	phosphodiesterase 6D, cGMP-specific, rod, delta
SENP1	1.29	6.98E-05	3.09E-02	SUMO1/sentrin specific peptidase 1
MIF	1.28	2.72E-05	2.02E-02	macrophage migration inhibitory factor
NCRNA00188	1.28	1.34E-04	3.78E-02	non-protein coding RNA 188
MRPS33	1.28	1.98E-04	4.52E-02	mitochondrial ribosomal protein S33
IFI27L2	1.27	9.80E-05	3.74E-02	interferon, alpha-inducible protein 27-like 2
ATP5H	1.27	3.10E-05	2.02E-02	ATP synthase, H+ transporting, mitochondrial Fo complex, subunit d
ZNF140	1.27	3.84E-05	2.13E-02	zinc finger protein 140
ATP5F1	1.27	2.29E-05	2.02E-02	ATP synthase, H+ transporting, mitochondrial Fo complex, subunit B1
SKP2	1.26	1.04E-04	3.78E-02	S-phase kinase-associated protein 2 (p45)
ZNF786	1.25	2.57E-05	2.02E-02	zinc finger protein 786
TUBB4Q	0.78	1.41E-04	3.85E-02	tubulin, beta polypeptide 4, member Q
PIM2	0.74	2.42E-04	4.91E-02	pim-2 oncogene

Significantly differentially expressed genes having a false discovery rates (FDRs, significance statistic adjusted for multiple testing) of ≤0.05 from an intensity-based Bayesian moderated t-test (IBMT) are shown. FC, fold change, calculated as the ratio of mRNA levels (athletes/controls)

### Identification of biological processes whose genes tend to be up- or down- regulated across the leukocyte genome in athletes

Differentially expressed genes were further analyzed using directional LRpath analysis. To capture the small, coordinated changes in gene expression that occur across a whole pathway, we used a less stringent significance level of *p* < 0.05 for gene selection and 2707 genes were selected as input. LRpath analysis revealed 57 GO BPs and 5 KEGG pathways significantly enriched (FDR ≤ 0.05). Among them, 52 were upregulated and 10 downregulated. Based on the semantic grouping of the enriched GO terms using ReviGO (with manual refinement), two super clusters were identified for upregulated biological processes including mitochondrial oxidative phosphorylation and gene translation (Table 3). Downregulated biological processes were centered on inflammatory responses. Other biological processes related to anti-apoptosis, gene transcription and regulation of RNA metabolic process were also downregulated in athletes (Table 4).

**Table 3:** GO terms and KEGG pathways significantly enriched with genes showing higher transcript levels in blood leukocytes in athletes vs. controls

Name	No. of Genes	Odds Ratio	*P* Value	FDR
Cellular Oxidative Phosphorylation				
KEGG: Huntington's disease	13	26.20	0.0001	0.0007
KEGG: Oxidative phosphorylation	13	7.93	0.0027	0.0037
KEGG: Alzheimer's disease	14	6.86	0.0029	0.0037
KEGG: Parkinson's disease	15	3.30	0.0139	0.0139
GO BP: generation of precursor metabolites and energy	14	4.95	0.0044	0.0227
Gene Translation				
KEGG: Ribosome	27	5.19	0.0003	0.0008
GO BP: translation	47	2.69	0.0001	0.0073
GO BP: translational elongation	27	5.12	0.0001	0.0073
GO BP: RNA splicing	11	6.21	0.0058	0.0274
GO BP: RNA processing	25	2.30	0.0082	0.0329

Kyoto Encyclopedia of Genes and Genomes (KEGG) pathways and Gene Ontology (GO) terms having false discovery rates (FDRs, significance statistic adjusted for multiple testing) of ≤0.05 from logistic regression-based method (LRpath analysis) are shown. No. Genes indicates how many analyzed genes belong to each enriched category; *P* value indicates significance of enrichment testing by LRpath analysis

**Table 4:** GO terms significantly enriched with genes showing lower transcript levels in blood leukocytes in athletes vs. controls

Name	No. of Genes	Odds Ratio	*P* value	FDR
Response to stimuli (immune responses)				
response to biotic stimulus	25	0.43	0.0002	0.0073
response to organic substance	29	0.43	0.0001	0.0073
regulation of developmental process	19	0.39	0.0003	0.0073
response to other organism	23	0.43	0.0003	0.0073
response to external stimulus	30	0.52	0.0010	0.0124
response to endogenous stimulus	18	0.41	0.0010	0.0124
multi-organism process	40	0.58	0.0022	0.0162
reproductive process	16	0.38	0.0009	0.0124
blood vessel development	11	0.35	0.0034	0.0187
response to wounding	31	0.56	0.0034	0.0187
myeloid cell differentiation	11	0.38	0.0055	0.0274
defense response	32	0.59	0.0062	0.0279
response to virus	12	0.41	0.0063	0.0279
hemopoiesis	18	0.50	0.0067	0.0279
regulation of cell proliferation	21	0.47	0.0016	0.0157
regulation of cell differentiation	14	0.38	0.0018	0.0157
response to extracellular stimulus	11	0.42	0.0095	0.0366
leukocyte differentiation	10	0.42	0.0138	0.0492
positive regulation of cell proliferation	14	0.49	0.0142	0.0492
cellular response to chemical stimulus	16	0.52	0.0142	0.0492
regulation of multicellular organismal development	16	0.38	0.0008	0.0124
negative regulation of developmental process	10	0.34	0.0044	0.0227
response to hormone stimulus	16	0.41	0.0017	0.0157
response to steroid hormone stimulus	10	0.38	0.0078	0.0320
homeostatic process	24	0.50	0.0021	0.0162
Regulation of Apoptosis				
negative regulation of apoptosis	17	0.35	0.0003	0.0073
regulation of apoptosis	24	0.49	0.0014	0.0148
anti-apoptosis	11	0.31	0.0020	0.0162
transcription				
transcription from RNA polymerase II promoter	20	0.42	0.0006	0.0119
regulation of transcription from RNA polymerase II promoter	18	0.45	0.0022	0.0162
negative regulation of transcription	10	0.31	0.0031	0.0187
Regulation of cellular metabolic process				
positive regulation of cellular metabolic process	23	0.46	0.0009	0.0124
positive regulation of nucleobase, nucleoside, nucleotide and nucleic acid metabolic process	12	0.34	0.0020	0.0162
negative regulation of macromolecule biosynthetic process	12	0.37	0.0032	0.0187
negative regulation of nucleobase, nucleoside, nucleotide and nucleic acid metabolic process	10	0.31	0.0031	0.0187
positive regulation of nitrogen compound metabolic process	13	0.39	0.0028	0.0187
positive regulation of cellular biosynthetic process	14	0.44	0.0058	0.0274
positive regulation of RNA metabolic process	10	0.40	0.0099	0.0373
positive regulation of multicellular organismal process	10	0.41	0.0122	0.0453
negative regulation of cellular metabolic process	20	0.55	0.0139	0.0492

Gene Ontology (GO) terms having false discovery rates (FDRs, significance statistic adjusted for multiple testing) of ≤0.05 from logistic regression-based method (LRpath analysis) are shown. No. of Genes indicates how many analyzed genes belong to each enriched category. *P* value indicates significance of enrichment testing by LRpath analysis

#### Technical validation of cDNA microarray data

To confirm the validity of the microarray data, we randomly selected two upregulated (*HRH4, FC = 1.55, p = 0.029*; *MS4A1, FC = 1.60, p = 0.0004*) and two downregulated (*ANXA3, FC = 0.55, p = 0.004; SLC22A4, FC = 0.67, p = 0.003*) genes identified by microarray and analyzed them using RT-PCR. The results showed that consistent with microarray analysis, *HRH4* and *MS4A1* were upregulated in athletes (*HRH4*, FC = 1.68, *p* = 0.006; *MS4A1*, FC = 1.49, *p* = 0.031), and ANXA3 and *SLC22A4* were downregulated (*ANXA3*, FC = 0.45, *p* = 0.009; *SLC22A4*, FC = 0.50, *p* = 0.052).

## Discussion

We investigated the transcriptional changes in the complete genome of peripheral blood leukocytes in young endurance athletes as compared with non-athlete controls. Gene-level testing and pathway analysis revealed that genes involved in mitochondrial OXPHOS and gene translation and ribosomal protein synthesis were significantly up-regulated in endurance athletes as compared to their non-athletic counterparts. The pathway analysis also revealed that the biological processes linked to inflammation were downregulated in athletes.

We observed extensive moderate changes (transcript changes ≤2 fold) in the leukocyte transcriptome of athletes. These moderate changes were expected. In our previous work as well as the research by others, we observed changes in transcript abundance in response to acute exercise stimuli that are largely transient [9, 12, 13], and most of these changes return to basal levels within 48 h [12, 13]. It is conceivable that genomic expression adapts over time to a new steady-state level, with small differences in transcript abundance, as found in yeast cells subjected to various environmental changes [14]. The coordinated changes we observed, albeit moderate, in the transcription levels of multiple genes within a particular biological process or a signaling pathway, may be critical to the alteration of immunological state and immune function in highly trained individuals.

Blood, a fluid tissue functioning to connect the entire biological system at the physical level, expresses over 80% of the genes in the human genome. It has been found that the expression profiles of circulating blood cells contain a specific signature in response to various physiological, pathological and environmental changes [15, 16]. Overall, the findings from the present study support this notion. The upregulation of mitochondrial OXPHOS and ribosomal protein synthesis, and downregulation of inflammation, as a consequence of endurance exercise training, have been frequently reported in skeletal muscle [17–19] and adipose tissue [20]. Thus, the results of the present study support the idea that peripheral blood can serve as a surrogate tissue to assess the effect of exercise training on the whole system.

The alterations in the athlete’s leukocyte transcriptome may not only reflect cellular changes occurring in other tissue types, such as skeletal muscle and adipose tissue, but may also reflect alterations in immune function, since blood cells constitute the first line of the immune defense system [15]. In the present study, we found that genes implicated in the cellular translation machinery were consistently upregulated in athletes. This included genes involved in RNA processing (e.g., *SNORD14E, SNORD4B, MIR15B, SNHG12, NCRNA00188*), and ribosome biogenesis (e.g., *RPL10A, RPL21, RPS27, RPS19, RPLP0, RPL23*). Vigorous exercise exerts a heavy assault on the biological system of participants, such as alterations in energy substrates, accumulation of metabolites, increases in body temperature, and changes in neuro-endocrine activity, etc. Living cells, including blood cells, exposed to these environmental changes, may respond with activation of protein synthesis, and accordingly activation of transcription, pre-mRNA processing, alternative splicing, etc. It is conceivable that the upregulation of genes involved in these processes is part of the molecular basis associated with the adaptation to long-term exercise training. Based on our results, the leukocyte transcriptional profile suggests that endurance athletes have a higher translation capacity and thus, protein production rate. Presumably, a higher protein turnover rate should be linked to an improved immune function due to the replacement of defective proteins with newly synthesized functioning proteins. However, the evidence linking this transcriptional change to immune function is lacking. Interestingly, a downregulation of these pathways and the pathways related to mitochondrial OXPHOS, has been identified as a key feature of aging immune cells (i.e., immunosenescence) [21]. Therefore, the results of our study suggests that transcriptional upregulation of leukocyte mitochondrial OXPHOS and ribosomal protein synthesis may be implicated as a protective effect of endurance exercise on immunosenescence.

In the present study, genes involved in mitochondrial OXPHOS and biogenesis were upregulated in athletes. They included those encoding electron transport chain proteins (such as *UQCR10, COX4I1, NDUFA12, ATP5J, ATP5H*), and genes encoding mitochondria ribosomal proteins (such as *MRPL51, MRPL28, MRPS33*). A similar finding has been made previously, in which genes encoding enzymes in the oxidative cycle had an upregulation in blood leukocytes following six months of high volume endurance exercise training [22]. Research over the past several years provides evidence that mitochondria play a fundamental role in the innate immune response involved in pattern-recognition, anti-bacterial immunity and sterile inflammation [23, 24]. Further, leukocyte mitochondria dysfunction, manifested by a decrease in mitochondria O_2_ consumption and an increase in the production of reactive oxygen species, has been implicated in the pathology of various diseases such as neurodegenerative disease [25], insulin resistance [26], type II diabetes [27], and cancer [28]. Accordingly, the data from our study suggest that intense exercise training can augment individual innate immunity and resistance to certain types of diseases via upregulation of mitochondrial energetics in circulating leukocytes. It is also plausible that this transcriptional change in leukocytes reflects a low metabolic and inflammatory stress from the whole system in athletes as compared with non-athlete controls.

Consistent with the majority of the studies on endurance exercise and inflammation, the anti-inflammatory effect of chronic exercise training was reflected in the leukocyte transcriptional profile of athletes. This finding was revealed through pathway analysis. The inflammation-related pathways, such as response to endogenous/external stimulus, defense response, regulation of cell proliferation, were significantly enriched among genes showing downregulation in athletes. However, the downregulation did not reach the significance level of FDR < 0.05 based on a gene-level test. The genes driving the enrichment of inflammation-related biological processes included both pro- (*IL-8, IL-15*) and anti-inflammatory cytokines (*DUSP1*), chemotactic factors (*CXCL8, CXCL1, PROK2*), and factors related to leukocyte migration (*ACTA2, PLSCR1, IFITM3*). At the protein level, the circulating immunoglobulins A, G and M were not significantly different between athletes and controls, which is consistent with some studies, suggesting that in the resting state, the plasma immunoglobulin levels of athletes and non-athletes are very similar [3]. However, the anti-inflammatory factor, IL-1ra, was significantly lower in athletes. At the transcriptional level, *IL-1RN*, the gene encoding IL-1ra, was downregulated (fc = 0.82) in athletes, but did not reach significance (*p* = 0.12). These results are in agreement with a previous study [29] that reported a coordinated downregulation of pro- and anti-inflammatory cytokines (including IL-1ra) in chronically trained elite kayakers. A similar finding in former elite athletes suggested that the decrease of cytokines was associated with high volume of current leisure time physical activity [5]. In our study, other cytokines (TNF-α, IL-1β, IL-6, IL-10) were not detectable in most of our samples likely related to the use of frozen blood samples. Overall, the transcriptional downregulation of inflammatory pathways and decreased plasma levels of IL-1ra appears to indicate a depressed inflammatory status in athletes. Interestingly, the genes associated with antigen presentation, including *HLA-DPB*, *HLA-DPB1*, *HLA-DPB2*, *HLA-DQA1*, and *HLA-DRA,* were upregulated in athletes. Thus, it is plausible to suggest that chronic vigorous exercise training has an anti-inflammatory effect; however, the immune function, especially the adaptive immune function, is less likely to be affected if not improved.

The clinical importance of these transcriptional changes is hard to predict because of the complexity of the immune system and the redundancy of immune functions. Additionally, the post-transcriptional regulation of gene expression might shift the profile of the end product of proteins. Nevertheless, if the actual activation status of the peripheral blood does mirror the expression data, the results of the present study suggest that chronic intense exercise training might be a double-edged sword with respect to affecting one’s health. It adversely influences participants’ efficacy of wound healing and their resistance to minor infection [30]. It also positively reduces one’s risk for inflammation-associated chronic disease (such as cardiometabolic diseases) and autoimmune conditions.

The biological processes related to the regulation of apoptosis, transcription, and regulation of cellular metabolic process, were also enriched among the downregulated genes. However, the genes driving the enrichment of these processes significantly overlapped (>80%) with those responsible for the enrichment of inflammation-related pathways. Thus, they may not have specific implications towards the impact of exercise training on leukocytes.

In the present study, we chose to study young and healthy athletes to minimize the influence of potential confounding factors such as aging and disease, that is known to influence immune function [31]. Also, it is worth mentioning that to avoid the potential immune dysregulation associated with intensified training and excessive emotional stress [3] , thus to best mimic the general population who undergo intense endurance exercise training for health and fitness purposes, the athletes were in their regular training period and were not preparing for any competition in the following three months.

Our study has a few limitations. First, we used the whole blood and did not account for the influence of changes in peripheral leukocyte subpopulations on the transcription profile. However, considering that gene expression may be influenced by manipulation inherent to the sorting procedure and the focus of the study is the overall immune status of the peripheral blood leukocytes, we believe that involvement (or not) of minor shifts in leukocyte populations/subpopulations, would not influence the valuable biological information conveyed by the results of the study. Second, due to a limited sample size, we could not examine males and females separately. However, in the design, the athlete and the control group were matched for sex. Thus, we believe that the findings of the study are the common features in both females and males. The sex effect of immune function should be investigated in a focused study in the future.

## Conclusions

In conclusion, our data indicate that in young healthy individuals, high intensity endurance exercise training can chronically induce transcriptional changes in the peripheral blood leukocytes. The directional changes in the transcriptional profile of leukocytes suggest that exercise can induce an upregulation of genes involved in leukocyte protein production rate and mitochondria biogenesis, as well as a downregulation of inflammation. The findings of the study also provide support for the notion that peripheral blood can be used as a surrogate tissue to study the systemic effect of exercise training.

## Methods

### Subjects

Twelve well-trained young endurance-swimming athletes (six males and six females; age, 18.4 ± 1 years; BMI, 20.3 ± 1.82 kg/m^2^) volunteered to participate in the study. Twelve sex-, age- and BMI-matched individuals (age, 19.1 ± 1.1; BMI, 20.6 ± 2.0 kg/m^2^) without training history were included as controls. All participants were recruited from the Shanghai University of Sport, Shanghai, China. All the athletes were grouped as being ‘national level’, who had participated in national competitions (such as National Youth Swimming Championships) and were ranked in the top 16 in their sport discipline. They were all experienced athletes and had been engaged in training for 5 to ten years (on average 8.7 ± 2.5 years). To avoid the potential immune dysregulation associated with intensified training and excessive emotional stress, the study was conducted when the athletes were in their regular training period and were not preparing for any competition in the following three months. During this period, the training regime of the athletes consisted of 8.8 ± 2.2 h/week of exercise at high-intensity including both in-water exercise and various forms of conditioning exercises. To ensure that none of the athletes suffered from overtraining syndrome, the athletes were asked to complete a standardized overtraining questionnaire proposed by the French consensus group on overtraining (French Society for Sports Medicine, SFMS), and no sign of overtraining was detected in any individual athlete. The Chinese translation of the SFMS questionnaire was used in the study. The English translation of the SFMS questionnaire was included in the Additional file 1.

None of the sedentary controls reported to have engaged in any type of vigorous exercise or perform more than 100 min of light-moderate intensity weekly exercise. Characteristics of all subjects are presented in Table 1. None of the athletes suffered from acute or chronic diseases or reported taking medications or antioxidant supplements. The female participants of the study had regular menstrual cycles and reported no use of oral contraception, and a blood sample was collected from them during the luteal phase of their menstrual cycles. All subjects were non-smokers. Subjects were fully informed as to the purposes and risks of the study before voluntarily giving their written informed consent. The study was approved by the Shanghai University of Sport Research Ethics Committee according to the principles set forth in the Declaration of Helsinki of the World Medical Association.

### Blood sampling and circulating levels of cytokines and immunoglobulins

Venous blood samples (5 x 2.5 ml whole blood) were drawn from the antecubital vein at rest in a sitting position in mornings after an overnight fast (~10 h). The subjects were instructed to avoid any strenuous exercise 48 h preceding the blood draw, and no exercise the day before. The concentrations of cytokines IL-1β, IL-1ra, IL-6, IL-10 and TNF-αin plasma were determined by Magnetic Luminex Screening Assays, according to the manufacturer’s instructions (R&D systems, Minneapolis, MN). However, IL-1β, IL-6, IL-10 and TNF-αwere all below detectible levels in our subjects and were not included in the results. Serum levels of IgM, IgG and IgA were measured by nephelometry. The inter- and intro-assay coefficients of variation of these measures are 0.81% and 1.51% for IgM, 0.88%and 6.1% for IgG, and 2.54% and 4.09% for IgA.

### RNA isolation and microarray gene expression procedures

Total RNA from blood samples of all subjects was isolated using PaxGene Blood RNA Kit (Qiagen) according to the manufacturer protocols. RNA quality and integrity were assessed using Bioanalyzer 2100 (Agilent Technologies, Santa Clara, CA). Total RNA (250 ng) from each sample was reverse transcribed to complementary DNA (cDNA), followed by overnight in vitro transcription to generate complementary RNA (cRNA). Then, cRNA was reverse transcribed, and the 7.5 μg of sense cDNA were fragmented and labeled. The quality of cDNA and fragmented cDNA was assessed using Bioanalyzer 2100 (Agilent Technologies, Santa Clara, CA). Labeled and fragmented cDNA was hybridized onto human gene 1.1 ST array strips (Affymetrix, Inc, Santa Clara, CA). The reactions of hybridization, staining, scanning and imaging were performed on the Affymetrix Gene Atlas instrument according to the manufacturer’s protocol.

### Microarray data analysis

Microarray hybridizations were analyzed on the software platform R 3.1.0 with Bioconductor 2.14.0 [32]. Initially, the expression data from all chips were background corrected, quantile normalized and summarized with RMA (Robust Multichip Average) [33]. One control sample was removed due to poor quality. Differentially expressed genes were tested by using an intensity-based Bayesian moderated t-test (IBMT) [34], with proven advantages in accuracy and stability of variance estimation. The resulting *p*-values were corrected for multiple testing with Benjamini-Hochberg method [35]. The data discussed in this publication have been deposited in the National Center for Biotechnology Information (NCBI)’s Gene Expression Omnibus (GEO) and are accessible through GEO Series accession number [GEO:GSE68072].

### Reverse transcription and quantitative real-time PCR

To confirm the validity of the microarray data, we selected four genes identified in the microarray analysis as significantly up- (*HRH4* and *MS4A1*) and down-regulated (*ANXA3* and *SLC22A4*) for a further evaluation by real-time PCR. Two micrograms of total RNA were used for cDNA synthesis using random hexamers primers (Invitrogen-Life Technologies, CA, USA) and superscript II reverse transcriptase (Invitrogen). The PCR was performed using StepOnePlus Real Time System (Applied Biosystem Foster City, CA, USA). Target gene levels were normalized by the geometric means of two housekeeping genes: β-actin and GAPDH. All reactions were performed in duplicate. For each gene, the fold change was calculated using 2^-ΔΔCT^ method, normalizing the single values with the mean of the control group transcript levels.

### Functional enrichment testing

To gain insight into which biological processes or molecular signaling pathways are responsible for the observed changes in transcription induced by chronic intense exercise training (athletic training), we used the data generated from the IBMT, including Entrez gene IDs, p-values, and fold-changes for enrichment analysis. Enriched Gene Ontology (GO) Biological Process terms [36] and Kyoto Encyclopaedia of Genes and Genomes (KEGG; http://www.genome.jp/kegg/) pathways [37] were tested by LRpath [38], a logistic regression -based gene set enrichment method. LRpath relates the odds of gene set membership with the significance of differential expression (p values from IBMT). LRpath’s ability to implicate important biological pathways in high-throughput data has been well established. GO terms and KEGG pathways with an FDR of less than 0.05 were deemed significant. We used a directional LRpath test to distinguish between upregulated and downregulated groups. Redundant/overlapping GO terms were removed by clustering similar terms semantically with REVIGO [39]. We employed a semantic similarity (SimRel) cutoff of 0.7.

### Statistical analysis

Statistical analysis was performed using SAS. The Wilcoxon Rank Sum test was used to test significant differences between groups in characteristics. *P*-values <0.05 were considered statistically significant.

## Additional file

Additional file 1: Overtraining questionnaire. (PDF 85 kb)

## Abbreviations

FC: Fold change
FDR: False discovery rates
GO: Gene ontology
IBMT: Intensity-based Bayesian moderated t-test
IL-1ra: Interleukin 1 receptor antagonist
KEGG: Kyoto encyclopedia of genes and genomes
LRpath: Logistic regression-based method
OXPHOS: Mitochondrial oxidative phosphorylation
