# Supplementary material for: Immune adaptation to chronic intense exercise training: new microarray evidence
Source: BMC Genomics. 2017 Jan 5;18:29. doi: 10.1186/s12864-016-3388-5 (PMC5216585; doi:10.1186/s12864-016-3388-5)
Supplement: Additional file 1: — Overtraining questionnaire. (PDF 85 kb) [file 12864_2016_3388_MOESM1_ESM.pdf]

Overtraining questionnaire

1 - My level of sport performance/my state of form has decreased:	Yes	No
2 - I am not as attentive as before:	Yes	No
3 - My close friends think that my behaviour has changed:	Yes	No
4 - I have a sensation of oppression in my chest:	Yes	No
5 - My heart seems to beat faster:	Yes	No
6 - I have a lump in my throat:	Yes	No
7 - I have less appetite than before:	Yes	No
8 - I eat more:	Yes	No
9 - I do not sleep as well as before:	Yes	No
10 - I drowse and yawn in the daytime:	Yes	No
11 - The lapse of time between training sessions seems to me too short:	Yes	No
12 - My sexual appetence has decreased:	Yes	No
13 - My performances are poor:	Yes	No
14 - I frequently catch a cold:	Yes	No
15 - I have put on weight:	Yes	No
16 - I have memory problems:	Yes	No
17 - I often feel tired:	Yes	No
18 - I underestimate myself:	Yes	No
19 - I often have cramps, muscular pain:	Yes	No
20 - I suffer from headaches more frequently:	Yes	No
21 - I do no feel fit:	Yes	No
22 - I sometimes feel dizzy, on the point of fainting:	Yes	No
23 - I do no confide in others so easily:	Yes	No
24 - I am often seedy:	Yes	No
25 - I have a sore throat more often:	Yes	No
26 - I feel nervous, insecure, anxious:	Yes	No
27 - I do no bear training so well:	Yes	No
28 - At rest, my heart rate is faster than before:	Yes	No
29 - During exercise, my heart rate is faster than before:	Yes	No
30 - I often feel rotten:	Yes	No
31 - I get tired more easily:	Yes	No
32 - I often have digestive disorders:	Yes	No
33 - I feel like staying in bed:	Yes	No
34 - I am not so confident in myself:	Yes	No
35 - I get injured more easily:	Yes	No
36 - I have more difficulties in organizing my thoughts:	Yes	No
37 - I have more difficulties in concentrating in my sports activity:	Yes	No
38 - My sporting gestures are less precise, less skilful:	Yes	No
39 - I have lost force and aggressiveness:	Yes	No
40 - I feel as if I had no one to talk to:	Yes	No
41 - I sleep longer:	Yes	No
42 - I cough more often:	Yes	No
43 - I do not enjoy practicing my sports as much:	Yes	No
44 - I do not enjoy my leisure activities as much:	Yes	No
45 - I get irritated more easily:	Yes	No
46 - I am less efficient in my school or professional activity:	Yes	No
47 - People around me think that I have become less pleasant:	Yes	No
48 - Training seems harder and harder:	Yes	No
49 - It is my fault if my results are worse:	Yes	No
50 - My legs feel heavy:	Yes	No
51 - I lose my personal things more easily (wallet, keys, etc.):	Yes	No
52 - I am pessimistic, I have the blues:	Yes	No
53 - I have lost weight:	Yes	No
54 - My motivation, will and tenacity are weaker:	Yes	No
